# Malaria-related ideational factors and other correlates associated with intermittent preventive treatment among pregnant women in Madagascar

**DOI:** 10.1186/s12936-018-2308-3

**Published:** 2018-04-25

**Authors:** Grace N. Awantang, Stella O. Babalola, Hannah Koenker, Kathleen A. Fox, Michael Toso, Nan Lewicky

**Affiliations:** Johns Hopkins Center for Communication Programs, 111 Market Place, Suite 310, Baltimore, MD 21202 USA

**Keywords:** IPTp, SP, Correlates, Ideation, Determinants, Malaria, Madagascar, Pregnancy, Provider

## Abstract

**Background:**

The Malagasy Ministry of Health aimed to achieve 80% coverage of intermittent preventive treatment of malaria among pregnant women (IPTp) in targeted districts by 2015. However, IPTp coverage rates of have remained fairly static over the past few years.

**Methods:**

During a cross-sectional household survey, mothers of children under the age of 2 years were asked about their most recent pregnancy. The primary outcome of interest was a mother receiving two or more doses of sulfadoxine–pyrimethamine (SP) (IPTp2) during their last pregnancy, at least one of which was obtained from a health provider. Multilevel analysis was used to account for community-level factors. Correlates included exposure to communication messages, the number of antenatal care (ANC) visits made by the woman, her household wealth, and other sociodemographic characteristics.

**Results:**

Over one-tenth (11.7%) of women received two or more doses of SP, at least one of which was obtained during an ANC visit. Two-thirds (68.3%) of women who consulted a health provider but did not take IPTp attributed this to not being offered the medication by their health provider. The odds of a woman receiving IPTp2 varied with her knowledge, attitudes, and perceived social norms related to IPTp and ANC and exposure to malaria messages. General malaria ideation, specifically the perceived severity of and perceived susceptibility to malaria, however, was not associated with increased odds of receiving IPTp2. A large variation in the odds of receiving IPTp2 was due to community-level factors that the study did not examine.

**Conclusions:**

Health communication programmes should aim to improve IPTp/ANC-specific ideation, particularly the norms of seeking regular care during pregnancy and taking any prescribed medication. While ANC attendance is necessary, it was not sufficient to meet IPTp2 coverage. Women surveyed in Madagascar rely on health providers to prescribe SP according to national policy. At the same time, stock-outs prevent health providers from prescribing SP. The large observed community-level variation in IPTp2 coverage is likely due to supply-side factors, such as SP availability and health-provider ideation and practices.

## Background

In Madagascar, 88% of the population lives in an area of high malaria transmission [1]. Malaria infection is the eighth most frequent cause of morbidity at community health facilities across the country [2]. Madagascar’s malaria burden, particularly among pregnant women, is a health priority because of the health risks malaria infection poses for not only a pregnant woman, but also her fetus and newborn child. In areas of high malaria transmission, *Plasmodium falciparum*, which causes the vast majority of malaria infections in Madagascar [3], is associated with both maternal anaemia [4] and increased risk of low-birth weight of newborns and contributes to higher risk of infant mortality [5]. The World Health Organization (WHO) recommends that pregnant women in zones of stable transmission receive sulfadoxine–pyrimethamine (SP) as part of intermittent preventive treatment for malaria in pregnancy (IPTp) [6].

Since 2004, the National Malaria Control Programme [Programme National de la Lutte contre le Paludisme (PNLP)] has recommended that pregnant women take at least two doses of SP to prevent malaria (IPTp2) [2]. In 2013, the Malagasy Ministry of Public Health (MOPH) set a national target of achieving 80% coverage of IPTp2 in targeted districts by 2017; these districts being those with stable or seasonal malaria transmission [7]. The MOPH recommended that both doses of SP be administered for free under the supervision of health workers during antenatal care visits (ANC)—the first dose upon the first fetal movement and the second a month later [2]. Although the 2013 Malaria Indicator Survey (MIS) did not estimate ANC coverage, the 2008–2009 Demographic Health Survey (DHS) found that 86.3% of women made two or more ANC visits [8]. The 2013 MIS estimated IPTp2 coverage was lower, at 20.6% [3], among target districts. Health programmes in Madagascar and elsewhere have sought to improve IPTp2 coverage, although their success has not been consistent. Identifying the factors associated with IPTp2 coverage is crucial for designing better programmes to address IPTp2 provision and malaria-related maternal and child mortality and morbidity. Health communication programmes, in particular, can benefit from understanding what factors are associated with pregnant women receiving IPTp to ensure their messaging is relevant and reaching the intended audience.

As national policy recommends the provision of SP during ANC visits [2], these factors would be expected to also play a role in IPTp uptake. Most pregnant women who receive at least one dose of SP in Madagascar obtain the medication during an ANC visit [3]. Subsequently, uptake of IPTp is largely dependent on a woman making ANC visits; as a result, IPTp and ANC attendance share many of the same correlates [9]. The factors associated with IPTp2 uptake occur at various levels: health systems [2, 10], health providers [2, 9], the households in which pregnant women live [11–13], and individual-level factors [12, 14, 15]. However, the role that psychosocial factors play at the individual level, and their association with IPTp, have been examined less frequently in quantitative studies. In contrast, the effect of a woman’s psychosocial characteristics on ANC attendance has been well documented. A woman’s involvement in the decision that she seek antenatal care, relative to her husband’s involvement, and her need for money or transport [16–22], often influences whether or not she makes ANC visits. The way a future mother perceives the quality of available health care [15] and the reasons why she may hesitate to disclose her pregnancy early [23, 24] can also impact ANC attendance.

Psychosocial factors that influence a behaviour have been framed using the ideation model. Ideation encompasses social, cognitive, and emotional factors at the community and individual levels [25]. The ideation model draws on various existing behavioural change theories and suggests that ideational factors—such as knowledge attitudes, perceived risk, subjective norms, self-efficacy, personal advocacy, and interpersonal communication—can influence health behaviours [25]. While the ideation model has recently been applied to explain variations in bed net ownership in Tanzania [26, 27], to date, no studies in Madagascar have examined the statistical association between a woman’s ideation and IPTp uptake.

Research from sub-Saharan Africa has indicated that women are often unaware of the benefits of taking IPTp [15] and some perceive it as unsafe [15, 21]. A systematic meta-analysis of data from sub-Saharan Africa found that a woman’s malaria and IPTp knowledge were correlated with her likelihood of taking IPTp2 [15]. Similarly, women who knew that SP was taken to prevent malaria were almost two times more likely to take a dose of SP as instructed by a health provider [28]. The literature offers mixed conclusions as to whether side-effects, either perceived or experienced, affect a woman’s likelihood of taking SP during pregnancy to prevent malaria [28, 29]. Data from Tanzania showed that women who experienced side effects took SP at a similar rate as women who had not [28]. This paper is the first study to take a detailed look at how a woman’s ideational factors, among others, may influence IPTp2 in Madagascar.

## Methods

### Sampling

**Fig. 1:**
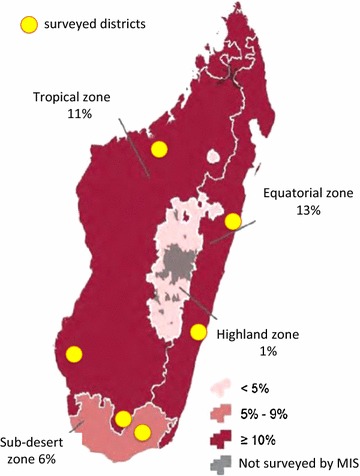
Prevalence of parasitemia by microscopy by malaria transmission zones (adapted from MIS 2013). The six surveyed districts represent three different transmission zones with varying parasitemia rates among children under 5 years old

Based on resource availability, six districts were selected at random from the districts in which MOPH recommends IPTp2. These districts included Marovoay, Morombe, and Bekily districts in the Tropical zone; Brickaville and Manakara districts in the Equatorial zone; and Ambovombe in the sub-desert zone (Fig. 1). A sampling frame in these six districts was obtained from the 2009 census conducted by the National Statistics Institute (*Institut National de la Statistique*). To maintain the safety of data collection teams, clusters in which security threats were known were excluded from the sampling frame. First, clusters were sampled in proportion to *fokontany* population size in order to select 90 clusters across the six districts—with one or two clusters being selected from each community or *fokontany*—the smallest administrative unit. Using this method, clusters with larger population sizes were more likely to be selected.

In the second stage of sampling, households within each cluster were selected using an interval calculated by dividing the number of households—from a complete listing of households enumerated in the cluster—by the number of households to be sampled in each cluster (20 households). Only households with children under five were eligible for participation. Although both the head of household and the female caregiver of a child under five were interviewed, this analysis focused on mothers of a child under the age of two. Among the 908 households, two households were surveyed in which the head of the household and the female caregiver were both mothers of a child under the age of two. Thus, the final sample included 910 adult women from 908 households (Table 1).

**Table 1:** Sample per district

District	Clusters	Eligible households	Mothers
Ambovombe	22	210	211
Bekily	8	55	55
Brickaville	10	99	99
Manakara	20	218	218
Morombe	12	120	120
Marovoay	18	206	207
Total	90	908	910

### Data collection

Data collection took place from September 14 to October 30, 2014. Interviewers used paper questionnaires and data entry clerks entered data twice in Epi Info (version 3.4.5). These data were then cross-checked for discrepancies and cleaned. The authors analysed the data using Stata (version 13.0).

### Variables

A binary variable indicated whether a woman with a child under 2 years old received IPTp2 during her pregnancy [30]. This was defined as her having taken at least two doses of SP [2], at least one of which she received during an ANC visit [30]. Each head of household described their household in terms of various physical characteristics, such as the type of roof, floor, and water infrastructure used. Principal component analysis was used to categorize each household as belonging to one of five wealth quintiles. Each quintile reflected the relative wealth of each household compared to all households sampled.

Each woman was also asked her age, religion, educational achievement, number of ANC visits, and from whom she had heard about IPTp. A woman’s level of exposure to malaria messages was measured with an additive index reflecting the number of sources from which she heard or saw malaria prevention and treatment messages during the past year. Three levels of exposure to communication sources were then defined based on this index.

Each woman responded to a set of questions to determine her general attitudes toward malaria that were modelled using an additive index ranging from 0 to 5 (Table 2). Although many questions were taken from the Roll Back Malaria (RBM) Malaria Behavior Change Communication Indicator Reference Guide [31], no standard set was used to measure ideation related to malaria. One point was given to a woman for each of the following: having talked to a spouse or friend about the risk of malaria within the past year, having basic knowledge about malaria, perceiving malaria as a serious illness, perceiving herself or her child to be susceptible to malaria, and being confident that she could prevent malaria. A woman was considered to have basic knowledge about malaria if she correctly identified the cause of malaria as mosquitoes, identified at least one effective way to prevent malaria and recalled fever as one of malaria’s symptoms. The remaining three ideational variables—perceived severity, perceived susceptibility, and self-efficacy to prevent malaria—were each measured using several Likert-scale questions from the RBM Guide. For example, women who agreed or strongly agreed that every case of malaria could potentially lead to death, received a score of 1 or 2, respectively. Conversely, women who agreed or strongly agreed that only weak children could die from malaria, received a score of − 1 or − 2. The scores from all Likert-scale questions related to a given ideational variable were summed to produce a net score. Women with a net positive score for perceived severity of malaria received one point on their general ideation index. In a similar manner, seven questions were used to identify women with net positive scores for perceived severity of malaria and three questions were used to identify women who were confident they could protect themselves or their children for malaria.

**Table 2:** Question items used to measure IPTp/ANC ideation among female caregivers

Ideational index and corresponding factors	Question/Likert-scale statement
General malaria ideation index	
Interpersonal communication about risk of malaria	In the past year, have you discussed malaria in pregnancy with your spouse or friends? *Yes*
Malaria knowledge	What causes malaria? Mosquito bites*
	What are some things that can happen to you when you have malaria? *Fever*
	What are the things that people can do to stop them from getting malaria? *Respondent mentioned at least one of prevention methods* (*e.g. sleep under a mosquito net*)
Perceived severity of malaria	I don’t worry about malaria because it can be easily treated*
	Every case of malaria can potentially lead to death*
	When my child has a fever, I almost always worry that it might be malaria*
	When someone I know gets malaria, I usually expect them to completely recover in a few days*
	When my child has a fever, I usually wait a couple of days before going to a health provider*
	My children are so healthy that they would be able to recover from a case of malaria*
	Only weak children can die from malaria*
Perceived susceptibility of malaria	During the rainy season, I worry almost every day that someone in my family will get malaria*
	People in this community only get malaria during rainy season*
	People only get malaria when there are lots of mosquitoes*
Self-efficacy to prevent malaria	I could probably easily protect myself from getting malaria*
	I could probably easily protect my children from getting malaria*
IPTp/ANC ideation index	
Descriptive norm	Generally, how many women in your community receive at least four check-ups from a health provider when they are pregnant? *All women, most women, at least half of the women*
Interpersonal communication	In the past year, have you discussed malaria in pregnancy with your spouse or friends? *Yes*
IPTp knowledge	What is the name of the medicine that is given to pregnant women to keep them from getting malaria? *SP/Fansidar/Paludar/Paludoxine*
	When should a pregnant woman start to take this medicine to keep from getting malaria? *When the baby first moves or at the start of the 4th month or second trimester*
	How many times during her pregnancy should a woman receive this medicine? *Two or more*
Attitudes toward IPTp/ANC	When a woman thinks she is pregnant, she should see a health provider as soon as possible*
	Pregnant women often feel sick when they take medicine on an empty stomach*
	Even if a woman thinks she may be pregnant, she should wait a few months to know for certain before she sees a health provider*
	Health care providers will only give a pregnant woman medicine if they know for certain that it is not harmful to her or to her baby*
Response efficacy	Pregnant women are still at risk for malaria even if they take the medicine that is meant to keep them from getting malaria*
Participation in health care decisions	In your household, who usually makes decisions about health care for yourself—you, your spouse, you and your spouse, or someone else? Respondent, joint decision with spouse*

The question responses that were considered in creating each index appear in *italics*

Statements without response options were responded to with a four-point Likert scale

*ANC* antenatal care, *IPTp* intermittent preventive treatment for malaria in pregnancy, *SP* sulfadoxine–pyrimethamine

Indicators marked with an asterisk (*) are from 2014 RBM Malaria Behavior Change Communication Indicator Reference Guide [31]

Each woman responded to a second set of questions to determine her ideation related to IPTp provided through ANC (IPTp/ANC, Table 2). A woman’s IPTp/ANC ideation was also modelled using an additive index ranging from 0 to 5. A woman received one index point for each of the following: perceiving that at least half the women in her community attended four or more ANC visits, discussing malaria in pregnancy with a spouse or friend in the last year, having basic knowledge of IPTp, being involved in making decisions about her own health, perceiving anti-malarial prophylaxis as effective in preventing malaria, having positive attitudes toward ANC care seeking, and taking such medication during pregnancy. A woman was considered to have basic knowledge of IPTp if she knew that SP was used to prevent malaria in pregnancy, knew when during pregnancy to start IPTp, or knew that she should take prophylaxis at least twice during her pregnancy. A woman’s attitude toward ANC and taking medication during pregnancy were measured by scoring her agreement or disagreement with four statements on a Likert scale from − 2 to 2. Women with a net positive score for these four questions received one point on their general ideation index.

### Analysis

The two-step analysis using bivariate and multivariate modelling; all analysis was restricted to data collected from mothers 18–49 years of age whose youngest child was less than 2 years old. Multilevel modelling was used to account for differences among communities (i.e. heterogeneity) that influence IPTp2 coverage. Multilevel analysis was run using Stata’s melogit command with individual women nested within sampling clusters. Results of an empty multivariate model predicting IPTp2 was used to determine the need for a multilevel analysis.

First, bivariate analysis was run to examine possible correlations between the primary outcome—having received two or more doses of SP, with at least one dose obtained from an ANC provider—with all possible covariates. Potential covariates included a woman’s individual sociodemographic characteristics, specifically her age, education level, religion, and marriage status (married or living as if married); ideational characteristics, such as general malaria and IPTp-specific ideation; frequency of media exposure, the number of sources from which she heard malaria prevention and treatment messages; the number of ANC visits at last pregnancy; characteristics of her household, particularly the mean household size, the number of children living in the household, and the household’s wealth quintile; and her district and malaria transmission zone. Those correlates that were significantly associated with IPTp2 in bivariate analysis were then included in the subsequent models.

Second, various multivariate models were run. Interaction terms were run to determine the significance of plausible correlations among covariates that were associated with IPTp2 coverage. Finally, a full multivariate model was created including any significant interaction terms from the previous step.

## Results

### Sample characteristics

Within the sampled households, the average household was composed of 5.3 individuals (Table 3), smaller than the national average household size (5.8, p < 0.001) [3]. The proportion of interviewed women were distributed unevenly across the six study districts, with the largest proportion (24.0%) coming from the Manakara district and the smallest portion coming from Bekily district (6.4%).

**Table 3:** Household and district characteristics of mothers, Madagascar 2014

	%/Mean (range)	n
Household		
Mean household size	5.3 (2–20)	910
Household wealth quintile		
Lowest	24.6%	224
Second	20.6%	187
Third	18.8%	171
Fourth	18.0%	164
Highest	18.0%	164
District		
Ambovombe	23.2%	211
Bekily	6.4%	55
Brickaville	10.9%	99
Manakara	24.0%	218
Morombe	23.2%	120
Marovoay	22.8%	207
Malaria transmission zone		
Sub-desert	23.2%	211
Tropical	42.0%	382
Equatorial	34.8%	317

Individual characteristics are displayed in Table 4. The women ranged in age from 18 to 48 years old and were 26.6 years old on average (not shown in Table 4). The sample was skewed toward younger women, which was similar to the national age distribution of similarly aged women who had had a child within the last 2 years [3]. As a whole, the study sample was less educated than similar women nationally [8]. For example, the proportion of women in the study sample who completed primary school (25.4%) was than about half of the national proportion among similar women (51.8%) [8]. In contrast, the study sample had a markedly higher proportion of women who identified with a traditional religion (23.6%) relative to the 2013 MIS (3.3%) [3]. This may be in part because the study question about religion did not allow respondents the option of “no religion,” which was how 30.8% of similar women described themselves in 2013 [3].

**Table 4:** Individual characteristics of mothers, Madagascar 2014

	%/Mean (range)	n
Sociodemographic characteristics		
Age (years)		
18–24	46.5%	423
25–34	36.7%	334
35–44	16.3%	148
44–50	0.6%	5
Educational level		
No formal school	35.2%	318
Some primary school	30.6%	277
Completed primary school	25.4%	230
Middle school or higher	8.8%	79
Religion		
Christian	68.4%	622
Traditional	23.6%	215
Muslim	1.4%	13
Other	6.6%	60
Married or living with partner	82.4%	740
Ideational characteristics		
General malaria ideation	3.0 (0–5)	910
IPTp/ANC ideation	1.8 (0–5)	910
Malaria message exposure		
Sources of exposure		
*Agent de santé*	25.7%	234
*Agent communautaire*	27.5%	250
Radio	23.7%	216
Family/friend	4.6%	42
Television	2.4%	29
Mean source index	1 (0–5)	n/a
Level of source exposure to malaria messages		
No exposure (0)	41.8%	380
Low exposure (1)	32.9%	299
High exposure (2–5)	25.4%	231
ANC attendance		
Number of ANC visits	3.4 (0–11)	910
No ANC visits	12.9%	117
One ANC visit	3.6%	33
Two ANC visits	8.7%	79
Three ANC visits	19.2%	175
Four or more ANC visits	55.6%	506

In terms of receiving preventive care during pregnancy, few women (14.1%) received two or more doses of SP and even fewer (11.7%) received two doses, of which at least one dose was received from an ANC provider (not shown in Table 4). Among the majority (89.4%) who consulted a health provider during their last pregnancy and did not take malaria prophylaxis during their last pregnancy, two-thirds (66.2%) attributed this to not being offered the medication by their health provider (not shown in Table 3).

### Bivariate analysis

Bivariate correlations were examined between women receiving IPTp2 and their individual characteristics and ANC attendance while adjusting for community-level variation. With regard to individual characteristics, analysis showed that a woman’s level of education, IPTp/ANC ideation index, and her level of exposure to sources of malarial prevention and treatment messages were all significantly and positively correlated with odds of receiving IPTp2. With regard to health-seeking behaviours, analysis showed that the more ANC visits a woman made during the pregnancy the more likely she was to receive IPTp2. A respondent’s place of residence was also associated with the likelihood of receiving IPTp2. These significant correlates were then used in multilevel analysis. Household size, household wealth quintile, the number of children under five in a household, and a woman’s age, religion, general malaria ideation index, and marital status were not significantly correlated with IPTp2 in bivariate analysis.

### Multilevel analysis

The empty or null model (Table 5, Model 1) nests women within communities to predict whether women will receive IPTp2. More than one-fifth (21.5%) of the total variance in IPTp2 was due to differences between clusters. This result supported the need for a multilevel model to estimate between-cluster variance by nesting women within clusters. The final model (Table 5, Model 2) indicated that a woman’s education level, the number of ANC visits she made, her IPTp/ANC ideation index, and the district in which she lived were significantly associated with the odds that she received IPTp2. Women who completed primary school were more than twice as likely to have received IPTp2 compared to similar women with no formal education (OR 2.13; 95% CI 1.12–4.10). Having completed a greater number of ANC visits was significantly and positively correlated with the odds of receiving IPTp2 (OR 1.26; 95% CI 1.10–1.45). A woman’s odds of receiving IPTp2 increased by 29% for every unit increase in her IPTp/ANC ideation index (OR 1.29; 95% CI 1.05–1.59), after adjusting for community-level factors and observed individual characteristics. Women who lived in Brickaville (OR 7.87; 95% CI 2.48–24.94), Ambovombe (OR 4.25; 95% CI 1.44–12.54), Morombe (OR 3.89; 95% CI 1.42–12.22), Bekily (OR 4.70; 95% CI 1.14–19.41), or Marovoay (OR 6.32; 95% CI 2.29–17.43) were all more likely to receive IPTp2 than their counterparts who lived in Manakara. The final model indicates that 17.4% of the variance in the likelihood of IPTp2 among sampled women was due to community-level factors.

**Table 5:** Multilevel correlates of IPTp2 in targeted districts, Madagascar 2014

	Model 1 OR (SE)	Model 2 OR (SE)
Fixed effects		
Individual factors		
Education level		
No formal school (RC)		1
Some primary school		1.05 (0.38)
Completed primary school		2.13 (0.71)*
Middle school or higher		1.03 (0.49)
IPTp/ANC ideation		1.29 (0.14)**
Level of source exposure		
No exposure (RC)		1
Low exposure		1.91 (0.55)*
High exposure		1.73 (0.55)
Number of ANC visits		1.26 (0.09)**
District		
Manakara (RC)		1
Brickaville		7.87 (4.63)***
Bekily		4.70 (3.40)*
Morombe		3.89 (2.27)*
Marovoay		6.32 (3.27)***
Ambovombe		4.25 (2.35)**
Random effects		
Cluster-level variance	0.90	0.69
Intraclass correlation	0.215	0.174
Log likelihood	− 318.06	− 287.20
AIC	640.1	602.4
n	910	904

Intermittent treatment of malaria in pregnancy is defined as two doses or more of SP, at least one of which was obtained during an ANC visit

*AIC* Akaikie’s Information Criterion, *ANC* antenatal care, *IPTp* intermittent preventive treatment for malaria in pregnancy, *OR* odds ratio, *RC* reference category, *SE* standard error, *SP* sulfadoxine–pyrimethamine

* p < 0.05, ** < 0.01, *** < 0.001

## Discussion

This paper has identified several significant correlates of IPTp2 uptake in six districts of Madagascar. These included a woman’s education level, her IPTp/ANC ideation index, the number of ANC visits she completed, her level of education, her district of residence and community-level factors. Although the study sample is not representative of all women of reproductive age in Madagascar or the six districts, it is the most comprehensive multivariate analysis of IPTp correlates in Madagascar to date. Only a few quantitative studies have examined the correlates of IPTp2 in Madagascar [12, 14, 32]. As far as the authors know, it is also the first study to investigate the association between a comprehensive list of ideational factors and IPTp2 in Madagascar’s targeted districts.

Unlike a meta-analysis from sub-Saharan Africa [32], the current study results did not confirm that women in areas of intermediate malaria prevalence were more likely to receive IPTp2 than those in low transmission areas. However, the current study’s sample was too small to make generalized statements about the differences in IPTp2 prevalence across different transmission zones, so the authors could not make conclusive statements about this correlate.

Based on relatively high general malaria ideation scores, mothers commonly perceive malaria as a severe disease, have basic knowledge about it, feel they are susceptible to it, are confident they can prevent it, and talk about the risk of malaria. The significant role of IPTp/ANC ideation, but not general malaria ideation, suggests that improving general attitudes and social norms regarding malaria is not sufficient to influence IPTp2 behaviour. Taking two doses of SP during pregnancy requires a sequence of health-seeking behaviours such as going to a health facility, taking medicine, and interaction with ANC providers. Consequently, it makes sense that different ideational factors were found to be associated with IPTp2, such as a woman’s involvement in the decision to seek preventive care and take any medication that she is offered. The significance of IPTp/ANC ideation as a correlate of IPTp2 uptake in the final model speaks to the important role of psychosocial factors, interpersonal communication, a woman’s role in decision making about her health, and her attitude toward ANC and taking medication during pregnancy in influencing IPTp2 uptake in Madagascar.

While this study did not directly investigate the role of supply-side factors, such as SP supply, there were several reasons to suspect that health-facility characteristics and health-provider practice accounted for the observed variation in IPTp2 coverage. First, most women who did not receive SP said it was because their ANC provider did not offer them malaria prophylaxis. Second, this study observed large regional differences in the odds of IPTp2 coverage that could be due to differences in women’s access to ANC, health-provider training, or the availability of SP. Third, community-level factors accounted for some of the variation of IPTp2 coverage, which may have reflected household distances to ANC providers or quality of ANC services. Literature from Madagascar [2, 9, 10] and sub-Saharan Africa [15, 18–21, 23, 32–34] indicate the crucial role of supply-side factors in determining IPTp coverage. Factors that reportedly limit IPTp2 coverage include a lack of recent IPTp health-provider training [18, 19, 35, 36], inadequate health-provider knowledge [17, 20], a lack of clear IPTp guidelines at health facilities [36, 37], and inconsistent availability of SP [35]. One qualitative study described a health provider in Madagascar who reported giving pregnant women two doses of SP a month instead of two doses during the entire pregnancy because the health provider felt the medication was “too weak” [10]. In Nigeria, one study found that while most health providers were aware an IPTp policy existed and what drug and how many doses to prescribe, they were not aware the national policy required directly observing women taking SP and did not know during what trimesters they should prescribe SP [17]. Such reports underscore the role of health-provider training and attitudes in ensuring IPTp is given according to national recommendations. In 2013, four out of five pregnant women in Madagascar, made at least two ANC visits but only one in five received IPTp2 [3]. It is plausible that the gap in IPTp2 coverage in Madagascar is not due to low ANC attendance, but is an unfortunate consequence of irregular SP availability or health-provider behaviours [32–38].

The positive correlation found between a woman making additional ANC visits and her odds of receiving IPTp is unsurprising, and consistent with other literature in sub-Saharan Africa [15, 32, 33]. The more ANC visits a woman attends, the more opportunities she has to receive two doses of SP.

While greater household wealth has been linked to increased odds of receiving IPTp2 in Madagascar [12] and in sub-Saharan Africa data [15, 32], this study found no such relationship after adjusting for a woman’s district of residence, number of ANC visits, malaria message exposure, IPTp/ANC ideation, education level, and community-level characteristics. This finding is similar to that of Kesteman et al. [14] who found no significant relationship between household wealth and a woman’s odds of receiving IPTp2. The absolute variation in wealth level among the rural households sampled may not have been sufficiently large, or household wealth may not influence IPTp2 coverage in Madagascar, at least partly, because of widespread availability of SP.

This study adds to a body of mixed evidence suggesting an association between a woman’s education and her likelihood of receiving IPTp. Like Clouston et al. [12], data showed that women who had completed primary school as their highest level of educational achievement had a significant advantage regarding their odds of receiving IPTp2 compared to women with no formal schooling. This was not a relationship observed in women attending sentinel health facilities across Madagascar [14]. Two meta-analyses of sub-Saharan Africa countries yielded contradictory findings concerning the role of educational achievement in predicting IPTp2 [15, 32].

Not all women in Madagascar make at least two visits to a health provider during pregnancy [3]. To address gaps in IPTp2 coverage, health communication programmes should improve IPTp and ANC-related ideation among women of reproductive age. Health communication programmes seeking to improve IPTp coverage should focus on IPTp-related ideation rather than general malaria ideation, such as one’s risk of malaria or the perceived severity of the disease. For example, programmes could encourage women to talk to their spouses or friends about the risk of malaria in pregnancy and promote early and complete ANC attendance as a community norm. Programmes could encourage families to include women in discussions about their care seeking during pregnancy. As the IPTp/ANC ideation index reflected women’s attitudes toward seeking ANC from health providers and health facilities, health programmes must address supply-side factors while maintaining demand for ANC services and trust in ANC health providers. Both health communication and health policy makers can address gaps in IPTp2 coverage by continuing to address supply-side barriers such as SP supply chain management and training for health providers to increase their IPTp-related knowledge and practices. Further research is needed to identify which health-provider ideational variables—such as knowledge and perceived efficacy of SP—need to be addressed in training and communication efforts. As the MOPH and PNLP continue to work toward higher IPTp2 coverage rates, they will require innovative ways to ensure SP availability at the health-facility level as well new mechanisms to train, supervise, and monitor health-provider provision of SP.

### Limitations

The study’s ability to describe correlates of IPTp2 is limited by the omission of supply-side factors from the study instruments. The data is not representative of the six districts surveyed or Madagascar as a whole. Because taking IPTp2 was self-reported by recently pregnant women, it is possible that actual rates of IPTp2 are higher than observed since few women or their partners in Madagascar know that malaria prophylaxis for pregnant women is available [9]. Women may not know what medication they are receiving, or, if they do, they might have forgotten the name of the medication or why it was given by the time of interview [10]. The analysis aimed to minimize recall bias among mothers by focusing on those whose youngest child was under 2 years. Although this study found a significant correlation between IPTp/ANC ideation and women receiving IPTp2, it measured ideational variables after women attended ANC and obtained IPTp, thus the analysis does not reflect a causal relationship between investigated factors and IPTp2. Lastly, findings may underestimate the role of exposure malaria messages because respondents were not asked about any specific malaria topic, message, or campaign.

## Conclusions

The results of this study suggest both supply-side and demand-side factors influence IPTp2 coverage in Madagascar. A woman’s individual ideational characteristics, such as her attitudes toward IPTp/ANC and her participation in the decision to seek care, are associated with whether or not she receives IPTp2. The authors suspect health-system factors account for much of the variance in IPTp2 coverage, confirming existing literature that points to the importance of health systems in ensuring access to quality IPTp services. Social and behaviour change communication programmes may be able to influence IPTp2 coverage by changing women’s attitudes specific to IPTp/ANC including gender norms that increase her involvement in making decisions about her health within a household and the importance of seeking ANC services regularly during pregnancy.

## Abbreviations

ANC: antenatal care
CCP: Johns Hopkins Center for Communication Programs
HC3: Health Communication Capacity Collaborative
IPTp: intermittent preventative treatment for pregnant women (with at least one dose of sulfadoxine–pyrimethamine)
IPTp2: intermittent preventative treatment in pregnancy, two doses (with at least two doses of sulfadoxine–pyrimethamine)
MOPH: Ministry of Public Health
PNLP: Programme National de Lutte contre le Paludisme
SP: sulfadoxine–pyrimethamine
WHO: World Health Organization
